# Modeling Environmental Tobacco Smoke (ETS) Infiltration in Low-Income Multifamily Housing before and after Building Energy Retrofits

**DOI:** 10.3390/ijerph13030327

**Published:** 2016-03-16

**Authors:** Maria Patricia Fabian, Sharon Kitman Lee, Lindsay Jean Underhill, Kimberly Vermeer, Gary Adamkiewicz, Jonathan Ian Levy

**Affiliations:** 1 Department of Environmental Health, Boston University School of Public Health. 715 Albany Street, Boston, MA 02118, USA; skmlee@bu.edu (S.K.L.); lju@bu.edu (L.J.U.); jonlevy@bu.edu (J.I.L.); 2 Department of Environmental Health, Harvard T. H. Chan School of Public Health. 401 Park Drive, Boston, MA 02115, USA; gadamkie@hsph.harvard.edu; 3 Urban Habitat Initiatives Inc., 328A Tremont Street, Boston, MA 02116, USA; kim.vermeer@urbanhabitatinitiatives.com

**Keywords:** smoking, environmental tobacco smoke, building simulation, indoor air quality, building retrofits, energy savings

## Abstract

Secondhand exposure to environmental tobacco smoke (ETS) in multifamily housing remains a health concern despite strong recommendations to implement non-smoking policies. Multiple studies have documented exposure to ETS in non-smoking units located in buildings with smoking units. However, characterizing the magnitude of ETS infiltration or measuring the impact of building interventions or resident behavior on ETS is challenging due to the complexities of multifamily buildings, which include variable resident behaviors and complex airflows between numerous shared compartments (e.g., adjacent apartments, common hallways, elevators, heating, ventilating and air conditioning (HVAC) systems, stack effect). In this study, building simulation models were used to characterize changes in ETS infiltration in a low income, multifamily apartment building in Boston which underwent extensive building renovations targeting energy savings. Results suggest that exterior wall air sealing can lead to increases in ETS infiltration across apartments, while compartmentalization can reduce infiltration. The magnitude and direction of ETS infiltration depends on apartment characteristics, including construction (*i.e.*, level and number of exterior walls), resident behavior (e.g., window opening, operation of localized exhaust fans), and seasonality. Although overall ETS concentrations and infiltration were reduced post energy-related building retrofits, these trends were not generalizable to all building units. Whole building smoke-free policies are the best approach to eliminate exposure to ETS in multifamily housing.

## 1. Background

Despite prevention and cessation campaigns, exposure to environmental tobacco smoke (ETS) remains a significant health concern, particularly in multifamily housing. ETS refers to a mixture of over 7000 organic and inorganic chemicals that are released into ambient air as gas or particles when exhaled from mainstream smoke, or produced as sidestream smoke from smoldering tobacco product. This mixture contributes to fine particulate matter (PM_2.5_: particulate matter less than 2.5 µm in diameter) in the indoor environment [1,2,3]. Studies have linked smoking and ETS exposure with pulmonary and cardiovascular diseases, liver, colorectal, and lung cancer, metabolic disorders, reproductive outcomes, such as low fertility, low birth weight and premature birth, sudden infant death syndrome, bone density loss, and tooth decay [3]. 

The home environment has been identified as the main source of ETS exposure [4,5]. In multifamily housing, ETS sources can include the resident smoker(s) as well as smokers living in other units within the building. In the US, it is estimated that of the 62.7 million people living in multifamily housing with smoke-free home rules, 44%–46% have experienced exposure to ETS infiltration [6]. A study in a Boston multifamily housing complex detected nicotine in up to 89% of nonsmoker units [7]. Other studies have documented reports of smoke intrusion or tobacco smoke smells in non-smoking apartments [7,8,9], and real-time instances of ETS transfer have been documented in smoke-free apartments from adjacent smoker occupied households [10]. King *et al.* found evidence of ETS PM_2.5_ movement from smoker to non-smoker units and shared hallway areas, with PM_2.5_ concentrations in smoker and non-smoker units ranging from 4.2 to 229.6 µg/m^3^ and from 1.6 to 15.7 µg/m^3^, respectively [8]. Identifying interventions to reduce exposure to PM_2.5_ from ETS in multifamily housing is critical to reduce the burden of smoking-related morbidity and mortality. However, it is challenging to characterize both the magnitude of ETS infiltration as well as the impact of interventions because of the complexity of airflow paths in multifamily buildings. ETS can move between apartment units through natural ventilation pathways (e.g., windows), mechanical ventilation systems, ductwork, shared walls, doors, and building cracks [11,12]. Building simulation models can be useful tools to study this complex behavior, and have increasingly been used to tease apart the effects of building interventions on indoor air quality [13,14,15].

In this study, we used a building simulation model to estimate the impact of building retrofits on the migration and infiltration of ETS across apartments in a 1960s-era multifamily housing complex located in Boston, MA, USA. In 2010, the housing complex underwent extensive energy-related building renovations, providing an opportunity to study changes in ETS migration in a real setting. Using building simulation models, infiltration of ETS PM_2.5_ was quantified across different units in a multifamily building and determined how ETS concentrations indoors were influenced by building interventions (e.g., weatherization, energy retrofits), human behavior (e.g., window opening, operation of localized exhaust fans), and seasonality. 

## 2. Materials and Methods

**Site**. Castle Square Apartments (CSA) is a 500-unit 100% low-income apartment complex located in Boston’s South End, which includes nineteen 4-story low rise buildings with 308 units (townhouses). The complex was constructed in the 1960s, when energy conservation was not considered in construction. In 2010, the property underwent extensive energy and green design improvements as part of a comprehensive 4% Low Income Housing Tax Exempt Bond rehabilitation, resulting in energy consumption reductions of 48% in the townhouse buildings. Building retrofits included air sealing, high efficiency mechanical improvements, window and door replacements, lighting and appliance replacements, and installation of bathroom and kitchen exhaust fans/systems. See Appendix Table A1 for renovation details.

**Figure 1:**
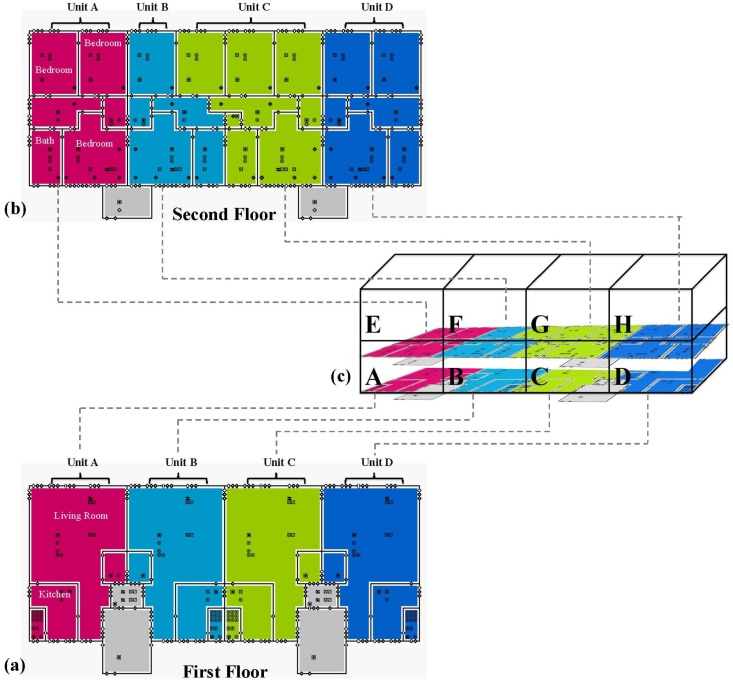
CONTAM building schematic representing the first and second floors of a typical 4-story townhouse building. The building has 8 townhouses total (4 across and 2 up), on a total of 4 floors. The figures present the floor layout for the: (**a**) 1st floor; and (**b**) 2nd floor of the building; and (**c**) the entire building. Colored zones represent each apartment. Dots inside the colored zones represent pollutant sources and sinks, floor airflow paths, zone identifiers, and air handling units, while dots on the walls represent airflow paths (e.g., windows, doors, frames, wall penetrations, exhaust fans).

**Building simulation.** CONTAM 3.1.0.3 (NIST, Gaithersburg, MD, USA) was used to simulate a typical CSA townhouse. CONTAM is a well validated multi-zone airflow and contaminant transport analysis model [16] that provides a framework for simulating building model construction and estimates airflows and contaminant concentrations of specified pollutants. The townhouse model template was developed based on architectural drawings, mechanical blueprints and construction specifications of a CSA townhouse building pre and post deep energy building retrofit. The units are organized as stacked duplexes, with the lower units on floors 1 and 2, and the upper units on floors 3 and 4. The most typical building configuration has 8 units: 4 units on floors 1 and 2, and 4 units on floors 3 and 4 (Figure 1). Each unit has a living room, kitchen, and mechanical room located on the lower level, and a bathroom and bedrooms (2 or 3) located on the upper level. Building dimensions, building elements (windows, doors), and mechanical ventilation systems were obtained from townhouse plans and blueprints, and used to assign room and building element dimensions. Leakage values were assigned to ceilings and walls (exterior, interior, and firewalls), wall joints (wall-ceiling, wall-floor), and window and door frames, kitchen dampers, and window air conditioning units based on effective leakage areas (ELAs) published by ASHRAE [17] or previous modeling studies. The ASHRAE tables publish minimum, average and maximum ELAs for building elements, and values for pre and post energy retrofit conditions were selected from this range. For elements not available from ASHRAE, leakage coefficients were selected from the building element with the closest matching description. No measurements were performed to verify the leakage values at CSA townhouses, thus the modeling results are representative of typical housing with CSA townhouse layout characteristics, but not the actual buildings. No information was available to validate the leakage values in the CSA townhouses, thus these leakage values are representative only of average building components. Appendix Table A2 shows the leakage values assigned per building element, by item, linear foot, or area. The building is heated via a forced air mechanical ventilation system which operated only during the heating season. Appendix Table A3 details the airflow rates and filter characteristics of the ventilation system. The stairwells were open to the outdoors and thus did not have a heating or ventilation system.

Indoor temperature profiles were assigned based on field measurements collected in a study conducted at 15 apartments in CSA (data unpublished). Hourly indoor temperatures were averaged across apartments to construct a 24-h indoor temperature schedule. On average, 24-h average indoor temperatures were 27 °C and 21.9 °C in the summer and winter, respectively. For outdoor meteorology, hourly values of solar radiation and weather conditions (e.g., temperature, relative humidity and direction) for a typical year were downloaded from the TMY2 dataset (Boston station 14739, 1961–1990: Typical Meteorological Year 2, National Solar Radiation Data Base, National Renewable Energy Laboratory). One January week and one July week were selected to represent typical winter and summer weather in the Northeast, respectively. In the winter, average outdoor temperature was −0.2 °C (range −9.4 to 13.9 °C), average relative humidity was 55.6% (range 25% to 96%), and average wind speed was 7.2 m/s (range 1.5 to 17.5 m/s). In the summer, average outdoor temperature was 23.7 °C (range 15.6 to 33.3 °C), average relative humidity was 63.8% (range 30% to 97%), and average wind speed was 5.5 m/s (range 0.5 to 9.8 m/s).

**Smoking parameterization.** One smoker was simulated in each townhouse unit in the building, and located in the living room. An emission rate of 10 mg/cigarette [18] and a deposition rate of −0.1/h [19] were assumed in the model. The smoker was assumed to be active from 7 a.m. to 7 p.m., and smoked a cigarette every half hour. Only PM_2.5_ from smoking was tracked and modeled in this analysis. PM_2.5_ from other sources such as cooking and outdoors were also modeled but are not discussed herein, they will be reported in a future publication. Predicted ETS PM_2.5_ from each smoker was tracked independently in order to differentiate between ETS PM_2.5_ from an in-unit smoker *versus* ETS PM_2.5_ infiltrating from other apartments.

**Multifactorial simulation.** Twelve multifactorial scenarios were simulated (Table 1) defined by building conditions before and after energy retrofit renovations, season (winter and summer), window opening (closed or open during cooking and shower events), and exhaust fan use (non-existent pre renovation, on/off post renovation). Windows were opened and kitchen exhaust fans were operated during stove operation periods only—10 min for breakfast and lunch, and 40 min for dinner. Bathroom exhaust fans were operated during showering events only. Flow rates for kitchen and bathroom exhaust fans were 160 cfm and 70 cfm, respectively, based on mechanical plan specifications. In all units interior doors between rooms were modeled as open all day, with the exception of the bedroom doors, which were closed during the night. Doors to the outside were opened for 1 min at 7 a.m., 9 a.m., 1 p.m., 5 p.m., and 7 p.m. every day. See Appendix Table A3 for details. All simulations were run during a typical winter and summer week (in January and July, respectively). In the winter, heating was regulated via a centralized mechanical ventilation system, which contained a Minimum Efficiency Reporting Value (MERV) filter, based on standards developed by ASHRAE [20,21]. The pre and post retrofit filters were assigned MERV ratings of 4 and 7, which approximately correspond to PM_2.5_ removal efficiencies of 10% and 40%, respectively. In the summer the centralized mechanical ventilation system was not operated, but window-mounted air conditioning units regulated indoor temperatures. The AC units are 100% recirculating and did not remove ETS PM_2.5_. Exhaust fans in the kitchen and bathroom were installed and operated post retrofit only, as no exhaust fans existed pre-retrofit. In order to isolate the impact of exhaust fans on infiltration, fans were only operated in units A and E for model simulations. 

**Statistical Analysis.** SimReadW (NIST, Gaithersburg, MD, USA) was used to convert CONTAM output files to text and Microsoft Excel files. SAS software (Version 9.2; SAS Institute Inc., Cary, NC, USA) was then used to calculate 24-h average ETS PM_2.5_ concentrations over 7 days each season, by apartment. Room volume was used to weight ETS PM_2.5_ concentrations and obtain an apartment average. Total infiltrated ETS PM_2.5_ was estimated by summing ETS PM_2.5_ from all other apartments measured in a specific unit, assuming there was a smoker in each unit of the building. 24-h average air exchange rates were also estimated for each apartment unit by summarizing the airflow across all paths (*i.e.*, windows, doors, cracks), and dividing by apartment volume. 

## 3. Results

Across all modeled units, seasons, window opening, and exhaust fan scenarios, 24-h average predicted concentrations of ETS PM_2.5_ generated from in-unit smokers present in all units ranged between 16 and 152 µg/m^3^ pre retrofit, and between 16 and 185 µg/m^3^ post retrofit. Predicted 24-haverage ETS PM_2.5_ concentrations due to infiltration from other apartments in the building ranged from 0.2 to 34 µg/m^3^ pre retrofit and 0.2 to 8.2 µg/m^3^ post retrofit. Note that these predictions assume there is one smoker in each apartment across the building. On average across all apartments and scenarios, 0.7% to 41% of the ETS PM_2.5_ concentration in a unit was due to infiltration from other apartments, and over 99% of this infiltration originated from directly adjacent apartments. In the winter, mean daily air exchange rates were 0.99/h (0.62–1.43/h) pre retrofit, and 0.79/h (0.49–1.18/h) post retrofit. In the summer, mean daily air exchange rates across all apartments and scenarios were 0.28/h (range: 0.16–0.49/h) pre retrofit, and 0.24/h (range: 0.13–0.50/h) post retrofit. 

**Figure 2:**
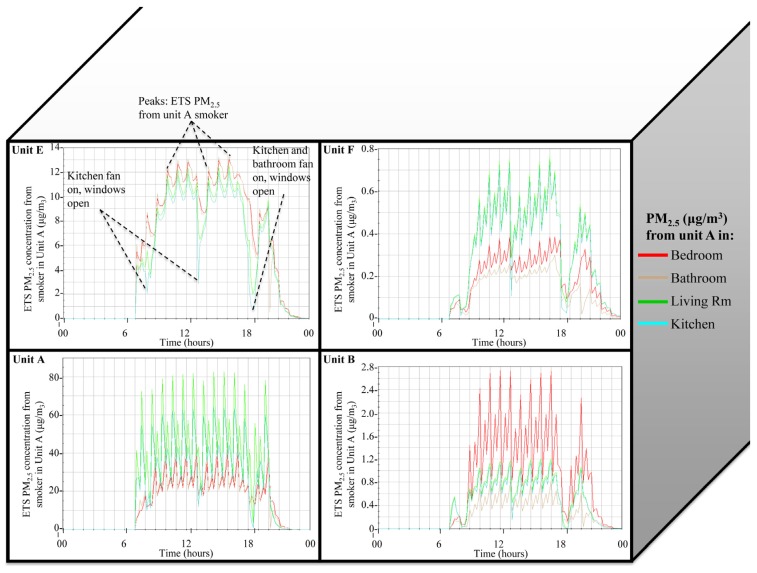
Predicted ETS PM_2.5_ originating from the smoker in unit A measured in the bedroom, bathroom, living room and kitchen of units A, B, E, and F over a 24-h period for a post retrofit model simulated during the winter, with windows open 3 times per day in all units, and kitchen and bathroom exhaust fan used during cooking and showering activities in units A and E only. PM_2.5_ concentrations in units B, E and F represent infiltration concentrations from unit A. The building is 2 townhouse units high and 4 units across, and only half the building is shown in the figure. A and E are end units, and B & F are interior units adjacent to units A and E (shown) and C and G (not shown). Note different y-axis scales.

Figure 2 is a compilation of annotated CONTAM output that illustrates the spatial distribution of predicted ETS PM_2.5_ generated by a smoker in apartment A across apartments A, B, E, and F, over time and in relation to occupant activities (e.g., window opening, kitchen and bathroom exhaust fans on). The figure is based on scenario 6 (post retrofit, winter, windows open, and kitchen and bathroom exhaust fan used in units A and E). The y-axis maximum is different for each graph, highlighting the magnitude differences (1–2 orders of magnitude lower) in ETS PM_2.5_ infiltration compared to primary generation, as expected. 

Table 2 summarizes the predicted 24-h average ETS PM_2.5_ concentration per apartment, averaged over a week, for ETS PM_2.5_ generated by the smoker in each unit as well as infiltrating from apartments located adjacent horizontally (right or left) and vertically (above or below). Results are stratified across all factors modeled. Pre retrofit, average ETS PM_2.5_ concentrations ranged between 22 and 33 µg/m^3^ in the winter and between 92 and 186 µg/m^3^ in the summer. Post retrofit average ETS PM_2.5_ concentrations ranged between 20–28 µg/m^3^ in the winter and 97–232 µg/m^3^ in the summer. Predicted concentrations were significantly lower in the winter compared to the summer, due to regular filtration by the mechanical heating system, fresh air introduced through the mechanical ventilation system, and the higher natural ventilation driven by indoor-outdoor temperature differentials in the winter. In the winter, filtration and increased fresh air supply reduced particle concentrations 40%. When the air handling system was off (and windows closed), particle concentrations were 64% lower in the winter compared to the summer due to the increased natural ventilation.

In the winter, retrofitting resulted in a decrease (~7%) in total predicted ETS PM_2.5_ concentrations in the downstairs units (A and B) but an increase (~4%) in the upstairs units (E & F). In the summer, retrofitting resulted in a concentration increase in all apartments (~22% on average) due to the increased air tightness with minimal changes in ventilation (*i.e.*, no change in fresh air supply and no filtration through the mechanical ventilation system). In the winter, vertical infiltration dominated due to the stack effect. Horizontal contributions of ETS PM_2.5_ were relatively small for all units, representing approximately 1 µg/m^3^ or less as an overall average. In the summer, vertical infiltration was observed in both the upper and lower level units, given daytime/nighttime temperature differentials that can change the direction of the stack effect. ETS PM_2.5_ concentrations and vertical infiltration were greater in interior units (B and F) compared to end units (A and E). Across both seasons, Unit B had consistently higher average weekly concentrations of ETS PM_2.5_ from the in-unit smoker, compared to the adjacent unit (A) and units located above (E and F), reflecting the influence of fewer outside walls/ceilings in reducing concentrations. Horizontal infiltration of PM_2.5_ was also greater in unit B compared to unit A, reflecting the influence of multiple adjacent units compared to other apartments.

In total, focusing just on infiltration (representing ETS PM_2.5_ exposure for a non-smoking household vertically and horizontally adjacent to units with smokers), retrofits led to a decrease in predicted ETS PM_2.5_ concentrations in the winter in units A, E, and F, but a small increase in unit B. As the ETS PM_2.5_ concentrations were greatest in the upper units due to the stack effect, the benefits in units E and F were far greater than the disbenefit in unit B. In contrast, in the summer, retrofits led to an increase in predicted ETS PM_2.5_ concentrations in all units, which were generally larger in magnitude than the concentration decreases estimated in the winter.

**Figure 3:**
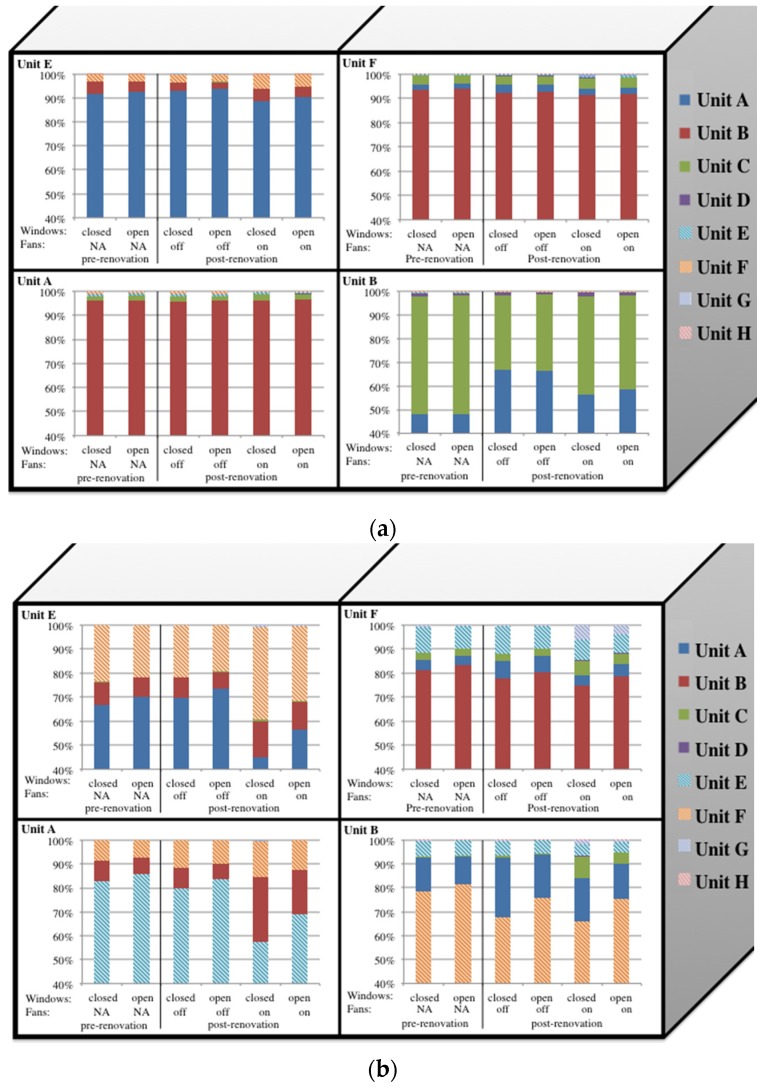
Percent ETS PM_2.5_ infiltrating from all other units into each apartment unit in the (**a**) winter, and (**b**) summer, across all simulated factors (windows open/closed, exhaust fans on/off, retrofit pre/post). In-unit ETS PM_2.5_ is not represented in this figure. Building is 2 units high and 4 units across, and only half the building is shown in the figure. A and E are end units, and B and F are interior units adjacent to units A and E (shown) and C and G (not shown). NA = not applicable as exhaust fans were not installed pre retrofit. Kitchen and bathroom exhaust fans were only operated in units A and E, so the influence of fans in units B and F reflect only fan activity in adjacent units.

For predicted ETS PM_2.5_ infiltration from other units, examining the relative contributions by unit provides insight about key infiltration pathways before and after retrofits (Figure 3). In the winter (Figure 3a), retrofits had a limited influence on all apartments except unit B, where the percentage infiltration from unit A increased and the percentage infiltration from unit C decreased. This was likely due to the air sealing that occurred between units B and C which did not occur between units A and B. Comparing pre and post retrofit infiltration in the summer (Figure 3b), changes in the origin of vertical and horizontal infiltration were observed, although with a strong dependence on window opening and exhaust fan use. When the exhaust fans were operated in units A and E, horizontal infiltration increased in both apartments, likely due to the change in pressure between apartments.

**Figure 4:**
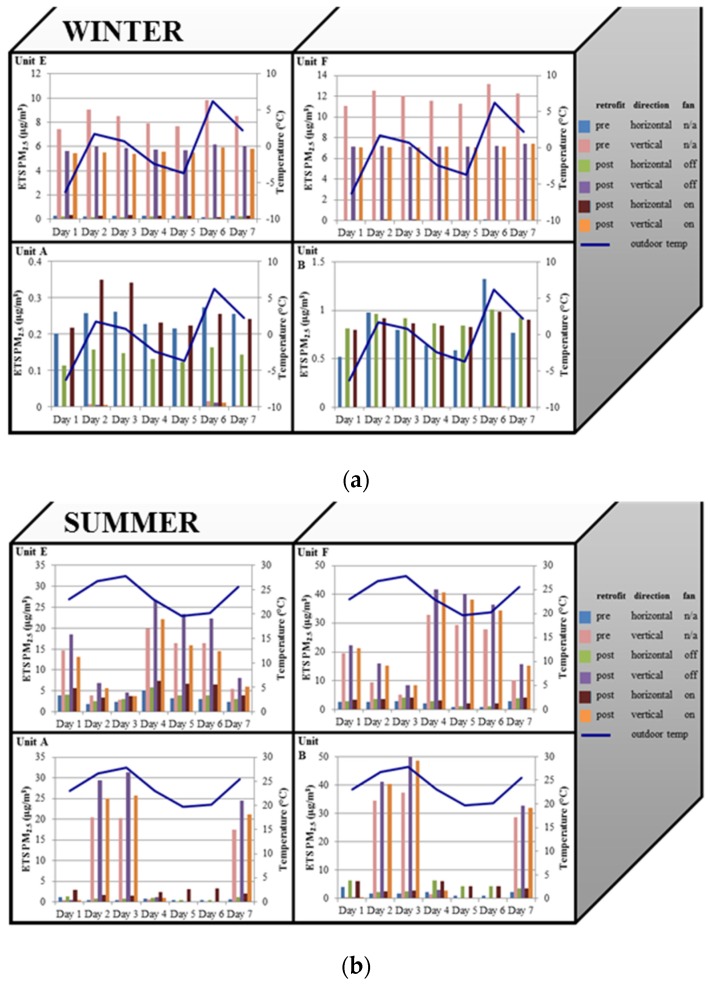
Predicted daily average ETS PM_2.5_ infiltration in A, B, E and F units from all other apartments during (**a**) the winter; and (**b**) the summer, over a week, compared to average daily outdoor temperature. Building is 2 townhouse units high and 4 units across, and only half the building is shown in the figure. A and E are end units, and B and F are interior units adjacent to units A and E. Note different maximum in y-axis scales.

Infiltration patterns are strongly influenced by daily ambient temperature, especially during the summer when ambient temperatures may fluctuate above and below indoor temperatures (Figure 4). On summer days when the average daily temperature outdoors was lower than indoors (days 1, 4, 5, 6), the infiltration magnitude was low (<10 µg/m^3^), with vertical infiltration contributing most to ETS PM_2.5_ concentrations in the upper units and horizontal infiltration contributing most in the lower units. On days 2, 3, and 7, a reverse stack effect occurred, and vertical infiltration increased by an order of magnitude in the lower apartments. In the winter, patterns were similar to the lower-temperature summer days, with vertical infiltration dominating, and outdoor air entering the downstairs apartment and diluting ETS PM_2.5_. In addition, in the upper units, increased outdoor/ambient temperature differentials led to increased vertical infiltration. 

## 4. Discussion

Across all units, seasons, window opening, and exhaust fan scenarios, the 24-h average predicted ETS PM_2.5_ concentration inside a smoking apartment across all apartments and scenarios was 73 µg/m^3^, ranging between 16 and 185 µg/m^3^. This estimate was for a smoker who smoked 24 cigarettes per day, with smokers in all adjacent units. These numbers are comparable to what has been modeled previously. In modeling studies, Chahine *et al.* estimated a mean concentration of 16.3 µg/m^3^ among households with smokers (average number of cigarettes smoked = 14.9) and a 99th percentile concentration of 67 µg/m^3^ [22], considering a range of home sizes and types, while Myatt *et al.* reported a mean concentration of 18 µg/m^3^ for households smoking eight cigarettes/day [23]. Comparing our predicted concentrations to those measured in field studies, a study of New York single family homes and efficiency apartments with indoor smokers, average ETS PM_2.5_ concentrations were 84 µg/m^3^ (range: 23–285 µg/m^3^) [24]. Dockery *et al.* found that one smoker adds 20 µg/m^3^ to PM_2.5_ personal exposure [25], and Ozkaynak found 30 µg/m^3^ higher concentrations in homes with smokers compared with non-smokers [26]. The lower concentrations reported in these last two studies compared to our models are likely due to differences in home volumes (larger), and smoking and ventilation rates. 

Predicted mean 24 hour average ETS PM_2.5_ concentrations infiltrating from other apartments in the building ranged from 0.2 to 34 µg/m^3^ across all scenarios. This infiltration concentration included ETS PM_2.5_ from all apartments, as a smoker was modeled in each unit in the building. However, over 99% of ETS PM_2.5_ originated from the apartments directly adjacent. Few published field studies have directly quantified the transport of ETS-related fine particles between smoking and non-smoking units. King *et al.* showed that median PM_2.5_ concentrations differed between non-smoking units, hallways and smoking units (8.3 *vs.* 16.6 *vs.* 20.2 µg/m^3^) [8]. In their study, PM_2.5_ concentrations in non-smoking units were similar to outdoor concentrations. Russo *et al.* enrolled smoking and non-smoking multifamily households in a study that collected real-time PM_2.5_ measurements (one-minute intervals), as well as diaries of smoking activity in the smoking households [10]. While this study was not designed to specifically estimate PM_2.5_ transfer, rough estimates can be made by comparing distributions of these data. For example, the 75th and 90th percentiles of PM_2.5_ concentrations in non-smoking units adjacent to smoking units were elevated during hours when smoking was reported, when compared to other hours (24.2 *vs.* 8.8 µg/m^3^ and 64.8 *vs.* 27.1 µg/m^3^). From these data, it could be inferred that these transfers are observable, and can easily exceed 30 µg/m^3^ over short time periods. Based on these studies, our modeled data are consistent with field-based measurements.

Air exchange rates in our study were much lower in the summer compared to the winter due to two mechanisms—the operation of the mechanical ventilation system with a fraction of fresh air supplied (fresh air was 5% of the total flow rate of 800 CFM), and the greater indoor-outdoor temperature differential which induces higher outdoor air infiltration. In the summer, the apartment units had a 100% recirculating air conditioning unit, which modified the temperature but didn‘t increase fresh air. Seasonal concentration patterns were also related to other model assumptions. For example, the window open schedule was based on cooking and showering activities, which were presumed to be equal in the summer and the winter. As has been published, window opening behavior is complex and can depend on many factors, including physical environmental, contextual, psychological, and physiological [27]. In reality residents open their windows more frequently in the summer, which results in higher summer air exchange rates [28]. In addition, there may be greater indoor smoking activity in the winter *versus* the summer. Because the same window opening schedule was maintained in the winter and summer for comparison purposes, winter air exchange rate in our study was higher as it was driven by greater temperature differentials. In Boston public housing units, higher PM_2.5_ concentrations in the winter were attributed to reduced winter air exchange rates and increased indoor smoking activity [29]. Our simulations were not meant to accurately characterize behaviors in each season, but to illustrate the joint influence of multiple factors (*i.e.*, indoor-outdoor temperature, pressurization, window opening) in a controlled experimental setting. Future work could include studying the impact of different window-opening schedules and capturing occupant behavior patterns that vary seasonally. 

One of the most important insights from our models is the strong dependence of ETS PM_2.5_ infiltration on apartment location within the building (*i.e.*, upper/lower level, interior/exterior status) and season. In the winter, ETS PM_2.5_ infiltration in upper level apartments (units E and F) was dominated by contributions from lower level apartments (units A and B). In contrast, lower level apartments (units A and B) had higher concentrations of ETS PM_2.5_ infiltrating from neighboring adjacent units. These findings are supported by Arku *et al.*, who also found higher PM_2.5_ concentrations on upper level areas during the winter [29]. In contrast, in the summer, ETS PM_2.5_ for units A and B predominantly originates from the units above (E and F, respectively), and a reverse stack effect leads to decreases in vertical infiltration to units E and F from units A and B, respectively. Coupled with our analyses of patterns in relation to daily average temperatures (Figure 4), this suggests that the stack effect (or reverse stack effect) plays a strong role in driving ETS PM_2.5_ movement in multifamily housing, even after air sealing retrofits. Results also reflect the importance of tracking weather conditions when conducting ETS PM_2.5_ field studies, as infiltration varies greatly depending on temperature differentials.

In the winter months, infiltration was reduced in units A, E, and F (with a greater reduction when kitchen and bathroom exhaust fans were operated in units A and E), with a modest increase in infiltration in unit B. Units A, E, and F each have at least three exterior surfaces that are exposed to the outdoors. In contrast, unit B has only two exterior walls, with units adjacent on two sides and above. The smaller number of exterior facing surfaces likely insulates unit B and decreases pressurization that occurs with differences between indoor-outdoor temperatures. Reduced exterior surfaces, reduce infiltration of cool air from the outside to the inside. While ETS PM_2.5_ is pressurized from unit A into unit B, air sealing reduces upwards movement of ETS PM_2.5_ that infiltrates from adjacent horizontal units, resulting in the increases of ETS PM_2.5_ observed. Although the goal of the energy retrofit was not unit compartmentalization, our results suggest that the minimal compartmentalization that was implemented (*i.e*., between-unit floor and ceiling caulking) resulted in modest reductions in ETS infiltration, although apartment location and season strongly influenced these effects.

Although previous studies have found moderate reductions (~30%) in inter-unit transfer of ETS from smoker to non-smoker units after increasing ventilation rates, balancing the mechanical ventilation system, and air sealing [30], our study results showed more limited changes with increases in the summer. The CSA retrofit included some attention to improved compartmentalization between apartments, primarily in the adjoining kitchen walls (between B and C, and F and G), but given the limited scope of this work in the retrofit we did not expect, and did not observe, much change in infiltration between A and B, and E and F.

There are some limitations to our approach. The CONTAM simulation was constrained to model a specific indoor temperature profile rather than dynamically interacting with the outdoor meteorology. CONTAM does not have a direct way of dynamically modeling indoor temperature, although it can link to a separate program with that capability. Because of the complexity of linking the two programs, the temperature profiles were applied based on field data collected from a previous field study at CSA. In addition, it can be difficult to model every exposure pathway for ETS transfer within occupied multifamily buildings. Door opening, thermal gradients, mechanical equipment operation, window opening and the location and timing of smoking activity are all relevant to these exposures. We have attempted to capture most of these drivers of exposure variability, and have captured random human activities such as door and window opening with a fixed schedule, which may not be representative of more random human behavior, which could contribute significantly to dilution and ETS transfer between units. As mentioned above, window opening was standardized to be the same in the summer and winter. Studies have shown that probabilistic modeling is a better way to model window opening behavior compared to standardized occupant behavior profiles [31]. However, since our objective was to compare infiltration pre and post retrofits, keeping a standardized schedule allowed us to make direct comparisons. Given our focus on multifamily housing, the small apartment volumes typical of subsidized housing, and the specificity of the townhouse building design based on details from CSA, the quantitative findings regarding ETS infiltration may not generalize to other types of homes. However, the general trends and drivers of infiltration are generalizable—for example the relative importance of stack effect *versus* horizontal infiltration. Another limitation is that the air leakage values are all uniform (e.g., only based on surface area) and are not based on measured values. Future studies should include detailed air leakage measurements to verify interior, exterior, and total air leakages pre- and post-retrofits. Although building a specific building template is time consuming, the analytical approach could be readily extended to other types of homes. Our results should also be interpreted with caution with respect to CSA itself, given that we did not try to capture the prevalence of different behaviors at CSA, but instead used our multi-factorial design to represent different types of units. Whether the deep energy retrofit led to net benefits or disbenefits depends greatly on the percent of smokers at CSA, where they are located in the building, and their window opening and other behaviors. That said, our model provides an indication of the types of units and behaviors where increases and decreases in ETS PM_2.5_ would be observed subsequent to an energy retrofit, and realistic occupant data can easily be integrated with our modeling platform and simulation outputs. 

This study illustrates the strength of building simulation tools to quantify indoor pollutant migration and track pollutant source contributions in complex buildings such as multifamily housing. Although retrofit and building modifications—including ventilation upgrades and air sealing—are common strategies to improve indoor environmental conditions, these strategies have limited usefulness in preventing exposure if smoke-free units are located in the same building as smoking units. In geographical areas with large seasonal temperature fluctuations, policies that sequester smoker units on a specific level may find increased or decreased ETS PM_2.5_ movement during specific seasons, due to the stack or reverse-stack effect. 

## 5. Conclusions

Model results from a multifamily apartment complex in Boston showed that although ETS PM_2.5_ infiltration from neighboring units is reduced after air sealing and ventilation improvements, pollutants generated from any given apartment can be detected in other units. Results provide evidence of the relative impact of housing characteristics (*i.e.*, apartment location), energy-related building retrofits, resident behavior (*i.e.*, window opening, exhaust fan use), and seasonality on ETS PM_2.5_ concentrations and infiltration patterns. Results support the body of literature suggesting whole building smoke-free policies are the only intervention that will prevent exposure to ETS for all residents in multifamily housing. Our study demonstrates the usefulness of building simulation models in quantifying the impact of building retrofits on pollutant infiltration in an existing apartment complex.

## Figures and Tables

**Table 1:** Multi-factorial scenarios of each model run representing changes in building retrofit status, season, window opening and exhaust fan use.

Scenario Elements	Pre Building Retrofits				Post Building Retrofits that the Data							
	Winter		Summer		Winter				Summer			
Scenario number	1	2	7	8	3	4	5	6	9	10	11	12
Windows ^a^	X	O	X	O	X	O	X	O	X	O	X	O
Bath and kitchen exhaust fans	Off ^b^	Off ^b^	Off ^b^	Off ^b^	Off	Off	On	On	Off	Off	On	On

^a^ Windows were open during cooking (3 times per day) and shower (3 times per day) events only. ^b^ Exhaust fans were only installed post retrofit, and operated in units A and E. X = windows closed; O = windows open.

**Table 2:** Predicted mean twenty-four hour average ETS PM_2.5_ concentrations generated by the smoker in each unit as well as the ETS PM_2.5_ concentration infiltrating from apartments located adjacent horizontally (right or left) and vertically (above or below). Results are stratified across all factors modeled (pre/post retrofit, window opening, exhaust fan use, season). Exhaust fans are only operated in units A and E.

Season	Pre Retrofit ETS PM_2.5_				Post Retrofit ETS PM_2.5_				Post Retrofit ETS PM_2.5_				Windows
	Kitchen and Bath Exhaust Fans OFF				Kitchen and Bath Exhaust Fans OFF				Kitchen and Bath Exhaust Fans ON				
	Unit	In-Unit Smoker	Horizontal Infiltration	Vertical Unfiltration	Unit	In-unit Smoker	Horizontal Infiltration	Vertical Infiltration	Unit	In-unit Smoker	Horizontal Infiltration	Vertical Infiltration	
		Mean (SD)	Mean (SD)	Mean (SD)		Mean (SD)	Mean (SD)	Mean (SD)		Mean (SD)	Mean (SD)	Mean (SD)	
		(µg/m^3^)	(µg/m^3^)	(µg/m^3^)		(µg/m^3^)	(µg/m^3^)	(µg/m^3^)		(µg/m^3^)	(µg/m^3^)	(µg/m^3^)	
**Winter**	***Scenario 1***				***Scenario 3***				***Scenario 5***				**Closed**
	A	23.0 (2.1)	0.3 (0.04)	0.01 (0.01)	A	22.2 (1.1)	0.18 (0.02)	0 (0.01)	A	20.5 (0.82)	0.36 (0.05)	0 (0.01)	
	B	29.4 (1.6)	0.91 (0.32)	0.01 (0.01)	B	26.0 (0.64)	1.0 (0.08)	0 (0.01)	B	25.8 (0.63)	0.99 (0.07)	0 (0.01)	
	E	17.3 (0.84)	0.3 (0.04)	9.3 (0.96)	E	18.6 (0.54)	0.23 (0.06)	6.5 (0.23)	E	17.1 (0.46)	0.38 (0.07)	6.1 (0.18)	
	F	19.3 (0.33)	0.06 (0.03)	13.5 (0.85)	F	19.5 (0.3)	0.06 (0.02)	8.1 (0.12)	F	19.4 (0.3)	0.12 (0.02)	8.0 (0.13)	
	***Scenario 2***				***Scenario 4***				***Scenario 6***				**Open**
	A	21.6 (1.9)	0.24 (0.03)	0 (0.01)	A	20.9 (1.0)	0.14 (0.02)	0 (0)	A	19.7 (0.84)	0.27 (0.06)	0 (0)	
	B	27.2 (1.4)	0.8 (0.27)	0.01 (0.01)	B	24.1 (0.61)	0.91 (0.07)	0 (0)	B	24.0 (0.61)	0.88 (0.06)	0 (0)	
	E	16.4 (0.79)	0.26 (0.03)	8.4 (0.84)	E	17.5 (0.52)	0.2 (0.05)	5.9 (0.2)	E	16.5 (0.49)	0.31 (0.06)	5.6 (0.2)	
	F	18.1 (0.33)	0.06 (0.03)	12.0 (0.74)	F	18.3 (0.28)	0.05 (0.01)	7.2 (0.11)	F	18.2 (0.28)	0.08 (0.02)	7.1 (0.12)	
**Summer**	***Scenario 7***				***Scenario 9***				***Scenario 11***				**Closed**
	A	96.0 (21.7)	0.95 (0.37)	10.5 (12.8)	A	121 (22.5)	1.5 (0.54)	15.6 (19.0)	A	91.5 (21.2)	4.7 (0.72)	12.4 (14.9)	
	B	141.9 (21.7)	3.3 (1.5)	19.3 (23.4)	B	185 (20.9)	7.8 (1.8)	22.6 (27.6)	B	180 (21.6)	8.0 (1.4)	21.2 (26.0)	
	E	105.0 (20.0)	4.9 (1.3)	15.7 (9.4)	E	129 (20.9)	6.5 (1.4)	23.5 (12.6)	E	96.8 (20.9)	9.8 (1.9)	15.1 (8.4)	
	F	151.6 (19.9)	4.0 (2.0)	30.2 (15.7)	F	180 (20.8)	6.0 (3.1)	45.2 (19.7)	F	173 (20.6)	7.2 (2.5)	42.4 (18.5)	
	***Scenario 8***				***Scenario 10***				***Scenario 12***				**Open**
	A	82.6 (18.4)	0.61 (0.28)	8.5 (10.3)	A	101 (18.8)	0.87 (0.35)	12.4 (15.1)	A	84.2 (19.6)	2.4 (0.71)	10.5 (12.8)	
	B	114 (17.7)	2.0 (1.0)	14.6 (17.8)	B	141 (16.3)	4.2 (1.6)	18.2 (22.2)	B	140 (16.9)	4.2 (1.4)	17.7 (21.6)	
	E	89.6 (17.2)	3.1 (1.1)	11.6 (7.0)	E	107 (17.5)	3.8 (1.1)	15.7 (9.0)	E	89.1 (19.2)	5.4 (1.6)	11.6 (6.7)	
	F	119 (16.3)	2.1 (0.95)	19.2 (11.1)	F	137 (16.2)	2.8 (1.3)	25.8 (13.4)	F	134 (16.7)	3.3 (0.81)	24.8 (12.8)	

## Appendix

**Table A1:** Summary of energy-saving building renovations conducted in the low-rise (townhouse) buildings at Castle Square Apartments in Boston, Massachusetts.

Renovation Element	Energy/Water Impact	Comfort/IEQ Impact
*Low-Rise Buildings (Townhouses)*		
New Windows: fiberglass dual pane windows. Flash openings and caulk to seal. Replace sliding glass doors with fiberglass French door and fixed glass.	• Reduce heating loads	• Reduce uncontrolled outdoor/indoor airflow • Reduce drafts
Unit Compartmentalization: reduce airflow between units by sealing penetrations in walls (especially kitchen walls and walls adjacent to mechanical closets) and installing thresholds and gaskets for unit entry doors.	• Reduce uncontrolled air movement; reduce exposure to combustion by-products from mechanical closets	• Reduce noise and odors from adjacent apartments
Ventilation: Install new kitchen and bath fans; add fresh air supply to heating system ductwork.	None	• Improve kitchen and bath exhaust—odor and moisture control • Improve air quality during heating season
Heating and Hot Water: Replace furnaces with boilers—install fan coils and re-use existing ductwork. Install indirect hot water tanks served by heating system boilers. Install new thermostats.	Reduce energy use for heating and hot water	• Improve comfort • Improve resident temperature control
Other: Low-flow fixtures, ENERGY STAR lighting and appliances	Reduce water use and energy use	None

**Table A2:** Building elements and corresponding leakage values used to construct the CONTAM townhouse building template pre and post a deep energy building retrofit.

Building Element	Pre-Renovation Condition			Post-Renovation Condition		
	*ASHRAE Element/Custom*	*Leakage Data*		*ASHRAE/Custom Element*	*Leakage Data*	
**Walls**						
exterior wall	custom	6.9	cm^2^/m^2^	custom	4.2	cm^2^/m^2^
interior wall	custom	2.0	cm^2^/m^2^	*no change*		
firewall general	custom	1.0	cm^2^/m^2^	*no change*		
firewall kitchen & bathroom	custom	1.0	cm^2^/m^2^	custom	0.8	cm^2^/m^2^
firewall mechanical room	WLEXMA_CAV	4.2	cm^2^/m^2^	custom	1.0	cm^2^/m^2^
**Doors**						
front & back doors	DRSINW_RMX	53.0	cm^2^/item	DRSIWE_RAV	12.0	cm^2^/item
front & back door frames	FRDRWDCA_RAV	0.3	cm^2^/m^2^	*no change*	0.3	cm^2^/m^2^
sliding glass door	DRSINW_RMX	53.0	cm^2^/item	DRSIWE_RAV	12.0	cm^2^/item
sliding glass door frame	FRDRMAUC_RAV	5.0	cm^2^/m^2^	FRDRMACA_RMN	0.3	cm^2^/m^2^
interior door frame	FRDR_RAV	12.0	cm^2^/item	*no change*		
mechanical room door	custom	12.0	cm^2^/item	*no change*		
mechanical room door frame	custom	0.3	cm^2^/m^2^	*no change*		
**Windows**						
windows	WNSLNW_RAV	1.1	cm^2^/m	WCCASEWE_RAV	0.2	cm^2^/m
window frames	FRWNWDCA_RMX	0.5	cm^2^/m^2^	FRWNWDCA_RAV	0.3	cm^2^/m^2^
Wall Joints						
ceiling-wall joint	JTCEWL_RAV	1.5	cm^2^/m	*no change*		
ceiling-wall joint (mechanical room)	JTCEWL_RMX	2.5	cm^2^/m	JTCEWL_RAV	1.5	cm^2^/m
ceiling-wall joint (kitchen)	JTCEWL_RAV	1.5	cm^2^/m	JTCEWL_RMN	0.2	cm^2^/m
floor-wall joint	JTFLWLUC_RAV	4.0	cm^2^/m	*no change*		
floor-wall joint (mechanical room)	JTFLWLUC_RMX	5.6	cm^2^/m	JTFLWLUC_RAV	4.0	cm^2^/m
floor-wall joint (kitchen)	JTFLWLUC_RAV	4.0	cm^2^/m	JTFLWLCA_RMX	1.2	cm^2^/m
**Wall Joints**						
ceiling	CE_RAV	1.8	cm^2^/m^2^	CE_RAV	1.8	cm^2^/m^2^
kitchen & bathroom fan damper	NONE PRESENT	--		custom	1.0	cm^2^/m
Air conditioning (AC), window units						
AC sleeve frame	FRWNWDCA_RMX	0.5	cm^2^/m^2^	FRWNWDCA_RAV	0.3	cm^2^/m^2^
AC leakage (summer)	custom	0.1	cm^2^/m^2^	*no change*		
AC leakage (off season)	custom	14.8	cm^2^/m^2^	custom	11.4	cm^2^/m^2^

**Table A3:** Characteristics and schedules for the mechanical heating system, exhaust fans, door and window opening.

Kitchen	Schedule
window open ^1^	10 min at 8:00 and 13:00, 40 min at 18:00. (same as cooking schedule)
exhaust fan on ^2^ *flow rate: 160 cfm*	
Bathroom	
window open ^1^	10 min at 6:00, 6:30, and 20:00 (weekdays) 10 min at 9:00, 9:30, and 20:00 (weekends) (same as showering schedule)
exhaust fan on ^2^ *flow rate: 70 cfm*	
Air Handling Unit	
supply and return fans on ^3,4^ *flow rate: 800 cfm* *fresh air: 40 cfm (5%)* *recirculating air: 760 cfm*	15 min per hour (all day)
Open Doors	
front and back ^1^	1 min at 7:00, 9:00, 13:00, 17:00, 19:00
mechanical room ^1^	1 min at 9:00

^1^ Air flow determined by two-way flow model in CONTAM; ^2^ Exhaust fans only active in post-retrofit scenarios; ^3^ Air handing unit supply and return fans only on in heating season (winter and spring); ^4^ Air handling unit uses MERV-4 and MERV-7 particulate filters in pre- and post-retrofit scenarios, respectively.
